# Lecture on Cholera

**Published:** 1885-07

**Authors:** Charles G. Raue

**Affiliations:** Phila.


### LECTURE ON CHOLERA.

CHARLES G. RAUE, M. D., PHILA.

(Delivered before the Nurse School of the Woman's Homœopathic Hospital, April 28th, 1885.)

Having been requested by the Nurse Committee of this Institution to deliver a lecture before you on "Nursing and Treatment in Cases of Cholera under Homœopathic Practice," I shall begin with telling you what Hahnemann had to say of cholera in the year 1831, fifty-four years ago. It is as follows: "Two opinions exactly opposed to each other prevail on the mode of propagation of the Asiatic cholera. One party considers the pestilence as only epidemic, of atmospheric-telluric nature, just as though it were merely diffused through the air, from which there would in that case be no protection. The other party denies this, and holds it to be communicable by contagion only, and propagated from one individual to another.

"Of these two opinions one only can be the right one, and that which is found to be the correct one will, like all truths, exercise a great influence on the welfare of mankind.

"The first of these opinions has the most obstinate defenders, who adduce the fact that when the cholera has broken out at one extremity of the town it may the very next morning be raging at the other extremity, consequently the infection can only be present in the air; and that they (the physicians) are in their own persons proofs of the non-contagious character of cholera, seeing that they generally remain unaffected by it and in good health, although they are daily in personal communication with those dying of cholera, and have even tasted the matter they ejected and the blood out of their veins, laid down in their beds, and so forth. This foolhardy, disgusting procedure they allege to be the *experimentum crusis*, that is to say, an incontrovertible proof of the non-contagious nature of cholera; that it is not propagated by contact, but is present in the atmosphere, and for this reason attacks individuals in widely distant places.

"A fearfully pernicious and totally false assertion.

"Were it the fact that this pestilential disease was uniformly distributed throughout the atmosphere like the influenza, then inexplicable would be the many cases reported by all the public journals where small towns and villages, during the prevalence of the murderous cholera, by the unanimous efforts of all their inhabitants, kept themselves strictly isolated, like a besieged fortress, and which refused to admit a single person from without, were exempt—inexplicable, I repeat, would be the perfect exemption of such places from the ravages of the cholera. This plague raged fiercely over an extensive tract on the banks of the Volga, but in the very middle of it, Sarepta, which had strictly and undeviatingly kept itself secluded, remained perfectly free from cholera, and up to a recent period none of the villages around Vienna, where the plague daily carries off a large number of victims, were invaded by cholera, the peasants of these villages having all sworn to kill any one who ventured near them, and even refusing to permit any of the inhabitants who had gone out of the villages to re-enter them. How could their exemption have been possible had the cholera been distributed throughout the atmosphere? And how easy it is to comprehend their freedom from it, seeing that they held aloof from contact with infected individuals.

"The course followed by the cholera in every place it traversed was almost uniformly this: That its fury showed itself most virulently and most rapidly fatal at the commencement of its invasion (evidently solely because at that time the miasm encountered none but unprepared systems, for which even the slightest cholera miasm was something new, never before experienced, and consequently extremely infectious), hence it then infected persons most frequently and most fatally.

"Thereafter the cases increased, and with them, at the same time, by communication of the inhabitants among each other, the quantity of diluted miasm. Thereby a kind of local sphere of cholera miasm exhalation was formed in the town, to which the more or less robust individuals had an opportunity of becoming gradually accustomed and hardened against, so that by degrees always fewer inhabitants were attacked or could be severely affected by it (the cholera was then said to take on a milder character), until at last the inhabitants were almost uniformly indurated against it, and thus the epidemic was extinguished in this town.

"It is a wonderfully benevolent arrangement of God that He made it possible for man to fortify himself against and render himself unsusceptible to the most deadly distempers, and especially the most fatal of them all, the infectious principle of cholera, if he gradually approaches it ever nearer and nearer, allowing intervals of time to elapse in order to recover himself, provided always he have an undebilitated body.

"*The cholera-miasm consists probably of innumerable invisible living beings in and about the patient.*"

Thus far I have cited Hahnemann's own words from a pamphlet published in Leipzig, 1831, translated by Dr. Dudgeon, of London, and incorporated in the lesser writings of Hahnemann, which were reprinted in New York, 1852.

It may interest you now to hear also something of the progress made in scientific researches since that plain statement of the case was written by Hahnemann, when cholera had appeared for the first time in Europe.

At least ten or twelve years ago it was already a generally accepted theory that the origin and development of cholera was due to parasites of the lowest form and smallest size; that these germs were transported by fluids and air, in which they were capable of living, and by different solid substances, to which they adhere, but that fluids were the most expedient vehicles, inasmuch as in them they could undergo considerable multiplication, and by them, when taken as a drink, could be transported to the very spot, the stomach and bowels, where they would at once multiply and cause the disease. For in many instances the spread of the disease had been traced to the use of water from certain pumps or rivers; and we find a large material of such observations in an article of Frankland "On the Water-supply of London and the Cholera" in the *Quarterly Journal of Science*, 1867.

Seeing that drinking-water had a most potent influence upon the dissemination of the disease, *Pettenkofer* commenced in the year 1854 his extensive and careful investigations as to the conditions of climate, weather, and underground water in their bearings upon cholera. His researches established the fact that the conditions of climate and weather had little or no influence, but that porous soils, which permit the penetration of moisture and fluids, were the very breeding beds for the spread of cholera-germs, especially if resting upon closely packed alluvial *clay*, which arrests the further escape of the underground water downward, and collects it all in no great depth under the surface. Any spring or well feeding off such ground-water must necessarily become contaminated and be the source from which cholera will be disseminated. This is in accordance with all the facts known. But this ground-water produces cholera only when the disease exists already *upon* the soil. It does not originate the seeds of cholera, but carries merely what it has received from the surface, and probably gives it a more or less favorable chance to multiply.

To this mode of propagation belong also the instances where wells and rivers become contaminated by privies or sewers containing cholera-germs. Even milk, if diluted with contaminated water or served in cans cleansed by such water, will have the same effect.

The *air*, too, is to a certain extent a carrier of the cholera-germs, especially in the immediate presence of a cholera patient, and when being long exposed to its influence. But that air can carry the poison over large tracts of land has never been proved, and Hahnemann already has refuted this idea.

Much more mischief is done by the adherence of the poison to *solid bodies*. Old clothing, linen, bedclothes, anything that has been soiled by the vomit or stools of the patient, will surely disseminate the disease. The instances where it has been imported to distant countries by old rags thus contaminated are not rare.

Thus far, then, the latest scientific researches correspond pretty closely with the views of Hahnemann which he entertained in regard to the spreading of the disease some fifty-four years ago, and although enlarged and more accurately defined by a large number of different observers, they are in essence the same.

Now, the question arises, What *is* the cholera poison? "The cholera miasm," said Hahnemann, "consists, probably, of innumerable invisible living beings in and about the patient." This was some fifty-four years ago. Some ten or twelve years ago, when the *germ theory* was brought vigorously into the foreground by microscopists to detect for every infectious disease a particular living organism—a microscopic fungus, known under the name of bacterium, microbe, or bacillus—it became a generally accepted theory that the origin and development of cholera also was due to parasites of the lowest form and smallest size. The cholera fungus, however, had evaded all detection until a year or two ago, when Dr. Koch, of Berlin, made his investigations in Egypt and the East Indies. He found, first in Egypt, in the stools of cholera patients a peculiar kind of microscopic fungi which he had not seen in the stools of any other bowel disease, and as he found the same again in the East Indies in cholera stools and in no other discharges from the bowels by other diseases, he came to the conclusion that these peculiar fungi must be the cholera germs so long sought after. He called them, from their resemblance to a *comma* used in writing, *comma bacilli*, and there is no doubt thus far that these comma bacilli are found only in cholera patients, especially in their stools—less frequently and much more sparsely in their vomit. As to whether they are the *cause* of cholera or a *product* or *concomitant* of the disease, is still the subject of lively contention among the doctors. And if doctors disagree, who shall decide?

We had better lay this question aside until the doctors do agree. But I tell you all this so that you may the better understand what concerns you most. The main question for us is undoubtedly this: *What shall we do if the pestilence should attack us?* For although Koch's work was a truly great one—a search after a deadly poison in a truly scientific spirit—he nevertheless did not finish it, because he did not find the remedy that would *cure;* he stopped short, so to say, in the midst of his sentence, with a *comma.*

Happily for us and those of the human race that will *heed* them, the efficient means to combat this dreadful disease *have* been discovered, used, and found not wanting in all the cholera epidemics since 1831. Hahnemann's great genius not only brought him, without the microscope—we might say intuitively—to the conclusion "that the cholera miasm consists, probably, of innumerable invisible living beings in and about the patient," but to finding out also the means and ways to *cure* the disease; he thus brought his sentence down to the *point.*

I will give you again what he has to say in his own words:

"Where the cholera first appears, it usually comes on in its first stage, with a tonic spasmodic character; the strength of the patient suddenly sinks; he cannot stand upright; his expression is altered; the eyes sunk in, the face bluish and icy cold, as also the hands, with coldness of the rest of the body; hopeless discouragement and anxiety, with dread of suffocation, is visible in his looks; half stupefied and insensible, he moans or cries in a hollow, hoarse tone of voice, without making any distinct complaints, except when asked; burning in the stomach and gullet and cramp-pain in the calves and other muscles; on touching the precordial region he cries out; he has no thirst, no sickness, no vomiting or purging.

"In this first stage *Camphor* gives rapid relief; but the patient's friends must themselves employ it, as this stage soon ends either in death or in the second stage, which is more difficult to be cured—and *not* with *Camphor*. In this first stage, accordingly, the patient must get as often as possible (at least every five minutes) a drop of spirit of Camphor (made with one ounce of Camphor to twelve of alcohol) on a lump of sugar or in a spoonful of water. Some spirit of Camphor must be taken in the hollow of the hand and rubbed into the skin of the arms, legs, and chest of the patient; he may also get a clyster of half a pint of warm water mingled with two full teaspoonfuls of spirit of Camphor, and from time to time some Camphor may be allowed to evaporate on a hot iron, so that if the mouth should be closed by trismus and he can swallow nothing, he may draw in enough of Camphor-vapor with his breath.

"The quicker all this is done at the onset of the first stage of the disease, the more rapidly and certainly will the patient recover; often in a couple of hours warmth, strength, consciousness, rest, sleep return, and he is saved.

"*It is, therefore, the members of a family alone that can most certainly and easily cure each other with Camphor spirit, because they are able instantaneously to aid those taken ill.*

"If this period of the commencement of the disease, so favorable to recovery and speedy cure by the above indicated employment of Camphor, has been neglected, then things look worse. Then Camphor is no longer serviceable. There are, moreover, cases of cholera, especially in northern regions, where this first stage, with its tonic spasmodic character, is hardly observable and the disease passes instantly into the second stage, with its chronic spasmodic character; frequent evacuations of watery fluid, mixed with whitish, yellowish, or reddish flakes, and along with insatiable thirst and loud rumbling in the belly; violent vomiting of large quantities of the same fluid, with increased agitation; groaning and yawning; icy coldness of the whole body, even of the tongue, and marbled blue appearance of the arms, hands, and face, with fixed, sunken eyes; diminution of all the senses; slow pulse; excessively painful cramps in the calves and spasms of the limbs. In such cases the administration of a drop of Camphor spirit every five minutes must only be continued so long as *decided* benefit is observable (which, with a remedy of such rapid action as Camphor, manifests itself within a quarter of an hour). If in such cases decided benefit is not soon perceived, then no time must be lost in administering the remedy for the second stage.

"The patient is to have one or two globules of the finest preparation of *Copper*, *Cuprum metallicum*$^{30}$, moistened with water, introduced into his mouth every hour or half hour until the vomiting and purging diminish and warmth and rest are restored. *But nothing else at all must be given;* no other medicine, no herb tea, no baths, no blisters, no fumigation, no venesection, etc., otherwise the remedy will be of no avail.

"Similiar good effects result from the administration of as small a portion of *White Hellebore* (*Veratrum album*$^{30}$), but the preparation of Copper is much to be preferred and is more serviceable, and sometimes a single dose is sufficient, which is allowed to act without a second being given as long as the patient's state goes on improving.

"The wishes of the patient of all kinds are only to be indulged in moderation."

As to the *prevention* of cholera Hahnemann says: "The above preparation of Copper, together with good, moderate diet and proper attention to cleanliness, is the most certain preventive and protective remedy. Those in health should take once every week a small globule of *Cupr.*$^{30}$, in the morning fasting, and not drink anything immediately afterward, but this should not be done until the cholera is in the locality itself or in the neighborhood.

"*Camphor* cannot preserve those in health from cholera, but only the above preparation of Copper; but when the latter is taken the vapor of Camphor must be avoided, as it suspends the action of the Copper."

This is Hahnemann's simple advice in his own words.

Now you will ask me, "How is it that we read in the papers of last summer that during the last epidemic in France and Italy there died fifty and more out of every hundred attacked, if cholera is so easily treated and cured?" True, so we did read, and so many did die, but they did not die under homœopathic treatment, and you must remember what I told you: *Hahnemann's advice can benefit only those who will heed it.* You will scarcely believe me, but it is a fact that *Dr. Rubini*, of Naples, a venerable old gentleman of eighty-four years, although he wrote long letters to the Mayor, the Prefect, the Cardinal, to all the proper authorities of Naples, to the King and his Secretaries, entreating them to follow Hahnemann's simple advice, and to put one-half of the cholera hospitals under the supervision of Homœopathy, and offered himself for active practice in those hospitals, never received an answer, save one from the Secretary, Depretis, to the effect "that he, the Secretary, could not interfere in medical matters, and that the use of Camphor and Homœopathy in the treatment of cholera patients belonged thereto."

And all such reckless heedlessness in the face of statistic facts, based upon authentic sources, and published in the daily newspapers of Naples, that in the nine cholera-epidemics which raged in Italy from the year 1838 to 1884, the allopathic school had lost in each at least fifty per cent., while the deaths under homœopathic treatment had amounted, even under the most unfavorable circumstances, to only from eight to twelve per cent. at highest! How is it possible, you will ask, that no notice was taken of such facts? Because, *for a time at least, preconceived ideas are stronger than the truth,* and if such ideas have taken possession of a governmental machine, this machine is bound to go by them, even if the world should perish. It was an idle and useless undertaking of Dr. Rubini, although prompted by the goodness of his heart, to appeal to such quarters. Already had Hahnemann complained in the following manner: "The physicians despise giving the simple, pure solution of Camphor because *I*, the reformer of the old injurious system of treatment, have recommended it from conviction, in the most urgent manner, in all countries of Europe." Well, never mind though; there is a still mightier power behind and above cliques and governmental machinery—*the will of the people*, after having received the truth—that will set it all right in time.

I shall give you now, in brief, from a direct report of Dr. *Tommaso Cigliano*, of Naples, sent to the *Allgemeine Hom. Zeitung* in Leipzig (Bd. 109, No. 20), what the homœopathic physicians did in Naples for the disinfection, prevention, and cure of cholera.

For *disinfection* they discarded entirely and altogether Carbolic acid, Chloride of Lime, Sulphate of Iron or Green Vitriol, Corrosive Sublimate and the like, and advised their patients to burn Sulphur in privies and dirty places and corners, in rooms where cholera patients had lived and articles remained which they had used. Persons who had been in contact with cholera patients were advised to expose themselves to the vapors of Camphor which had been allowed to evaporate on a hot iron, according to

Hahnemann's direction. The dejections of cholera patients were mixed with a few spoonfuls of spirit of Camphor.

As *prophylaxis* they recommended *Rubini's* solution of Camphor, which is made of one hundred parts of Camphor to one hundred parts of spirits of wine; they advised it in doses of five, three, or two drops twice or three times a day. By these means they achieved marvelous results.

Of the two thousand families who were under their regular medical care; of one hundred working men in the typographical institute of De Angelis, with their families, amounting to five hundred or six hundred persons more, and living in the most affected parts of the city, and of the inmates of the Central House of the Sisters of Mercy of five hundred to six hundred persons, not *one* person was attacked by the cholera! All had been using Rubini's solution of Camphor, as above described.

In *treating* cholera patients they relied almost exclusively on Rubini's Camphor-solution, giving four or five drops on sugar, or more in bad cases, every ten to fifteen minutes, until reaction set in, when the doses were decreased and given in longer intervals. They used also Veratrum and Cuprum and other remedies with good results, but their mainstay was Camphor for all stages.

As *drink*, they allowed fresh water, a little at a time, sometimes with a few drops of rum or cognac. If water would not stay on the stomach they ordered small pieces of ice, although this only rarely.

As *nourishment*, they allowed, after perspiration and vomiting had ceased, meat broth, or water soup, or bouillon soup, also a little watered wine. They never allowed any interference with any other drug, nor baths of any kind, nor change of bed or clothing until after the perspiration had ceased.

Their losses were about one in a hundred.

This is from the direct report of Dr. Tommaso Cigliano's own personal observations during last year's cholera epidemic at Naples, and it seems to me that its treatment might satisfy even the materialistic tendencies of the allopathic school, if they could conquer their equally strong proclivities of mixing. Laudanum, Morphine injections, baths, and other adjuvants will never do with the administration of Camphor nor without it.

I must, however, here put in a word of warning against overdosing with Camphor. Camphor in an over-dose will do harm, and may even kill as well as cholera, and in a very similar manner, and herein lies its homœopathicity to cholera. If when taken as a preventive or during reconvalescence it causes stupefaction, somnolence, precordial anxiety, coldness and bluishness of the skin, its use must be discontinued or the dose greatly diminished.

The best way of administering Camphor is on pieces of sugar; in water it causes nausea and loathing to such an extent that patients refuse to take it.

For injections it is best applied in oil or in warm water with some alcohol.

Another prophylactic remedy which Dr. C. Hering recommended years ago, and which has proved equally successful in the several cholera epidemics of this country, is this: Take pulverized or *precipitated Sulphur* and put a pinch of it into each stocking or shoe you are wearing; renew about twice a week. The Sulphur will be absorbed by the skin and disinfect the body of any cholera germs that might chance to enter it, just as burning Sulphur will disinfect rooms or clothing.

The linen, bed sheetings, etc., should always, before they are given to the washerwoman, be thoroughly disinfected by exposure to the fumes of burning Sulphur in a suitable room.

As regards public sanitary measures, such as the removal of filth from the streets, the abatement of nuisances, the filling up of wells in the city which may become contaminated by privies, sewers, or ground-water, the purifying of our rivers from which we draw our drinking-water, the prevention of importing the cholera germs from abroad, I have nothing to say. These are matters belonging to the proper authorities, and it is only fair to say that they seem to be fully awake to the danger and conscious of their duties, and do the best they can.

As there is nothing more detrimental to health in cases of epidemics than vague fear, the feeling of uncertainty and helplessness as to how to face a danger, resulting mostly in a panic, I hope that by these explanations I may have dispelled any such vague fear from your minds in having stated to you clearly not only the danger of cholera, but also the ways and means by which it can be prevented and cured. Look out, then, with confidence and courage into the future, trust in God, and keep your Camphor ready!

---

Symptoms of Washing.—*Sulphur*, aversion to washing (also *Antimon. c.* and *Ammon. carb.*); *Borax*, washing chest with cold water relieves chest symptoms; *Antimon. crud.*, child cries when washed in cold water; better when washed in warm water; *Nat. mur.*, desire to wash in cold water; *Æsculus*, after washing hands and face swell enormously and become red; *Thuja*, skin of face peels off when washed; *Acon.* and *Fluor. ac.*, better from washing.

